# Supplementary material for: MiR193a Modulation and Podocyte Phenotype
Source: Cells. 2020 Apr 17;9(4):1004. doi: 10.3390/cells9041004 (PMC7226544; doi:10.3390/cells9041004)
Supplement: Supplementary file 1 [file cells-09-01004-s001.pdf]

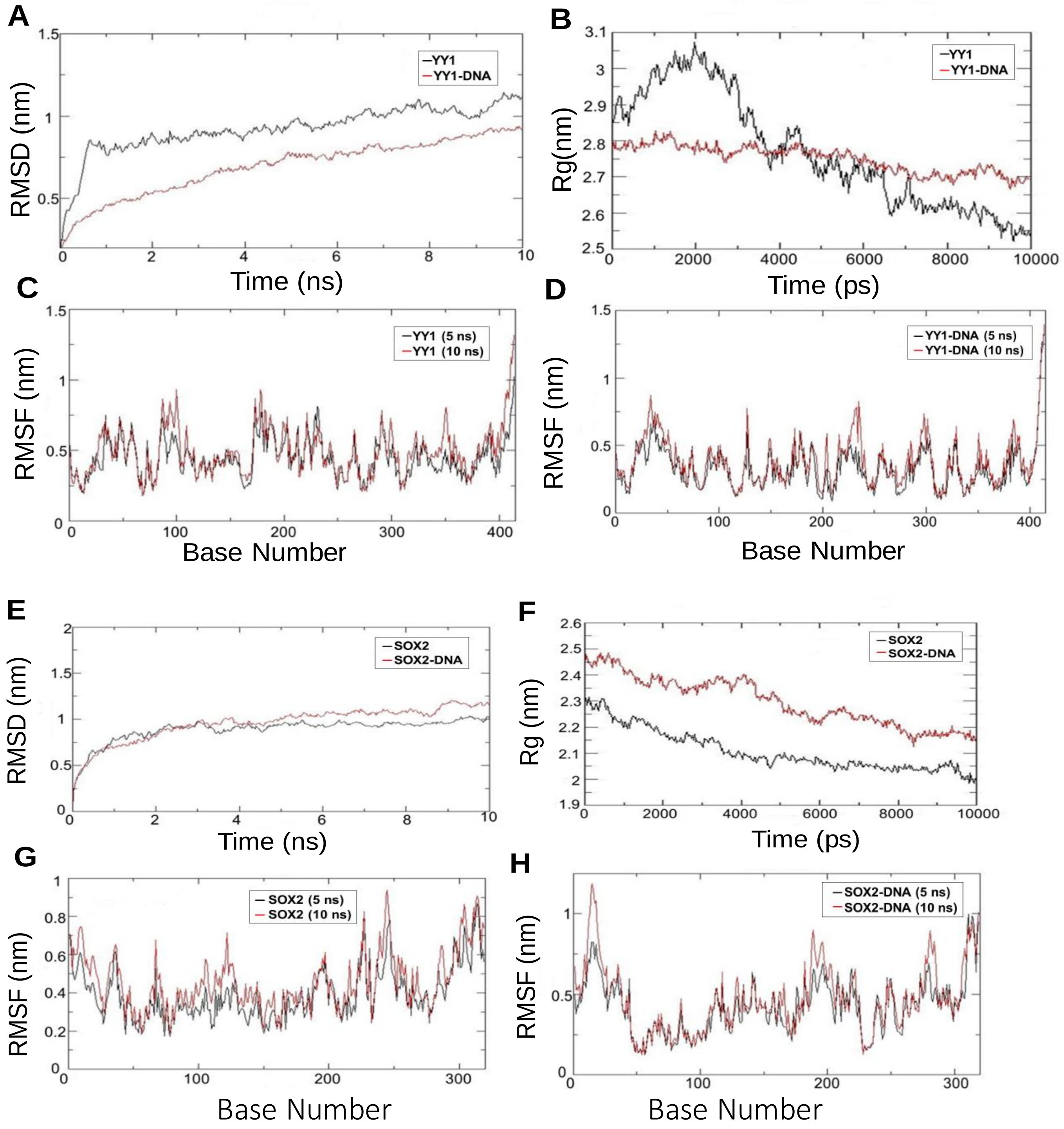
A
RMSD (nm)
Time (ns)
YY1
YY1-DNA
B
Rg(nm)
Time (ps)
YY1
YY1-DNA
C
RMSF (nm)
Base Number
YY1 (5 ns)
YY1 (10 ns)
D
RMSF (nm)
Base Number
YY1-DNA (5 ns)
YY1-DNA (10 ns)
E
RMSD (nm)
Time (ns)
SOX2
SOX2-DNA
F
Rg (nm)
Time (ps)
SOX2
SOX2-DNA
G
RMSF (nm)
Base Number
SOX2 (5 ns)
SOX2 (10 ns)
H
RMSF (nm)
Base Number
SOX2-DNA (5 ns)
SOX2-DNA (10 ns)

Supple. Fig.1

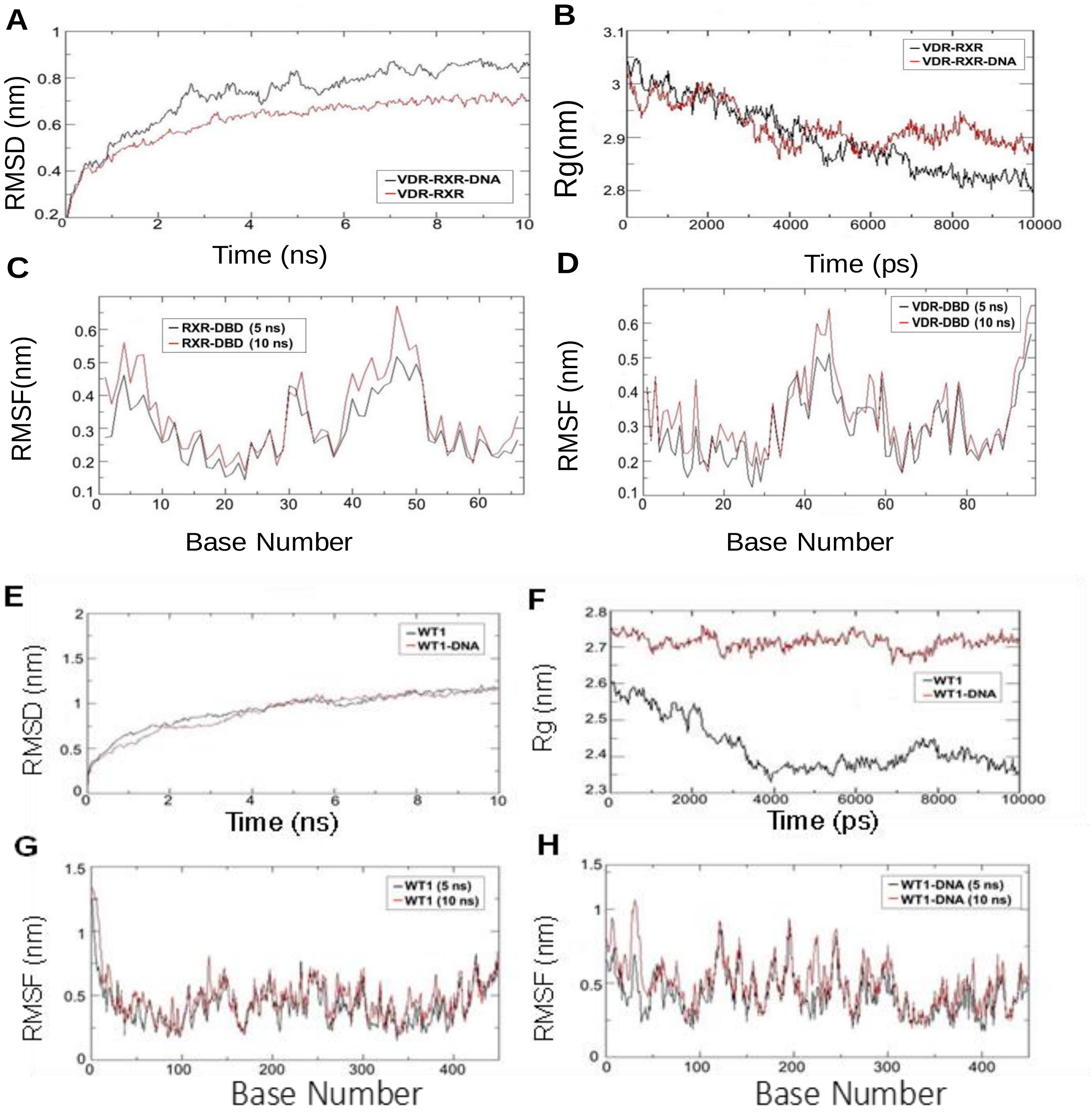
A
RMSD (nm)
Time (ns)
VDR-RXR-DNA
VDR-RXR
B
Rg(nm)
Time (ps)
VDR-RXR
VDR-RXR-DNA
C
RMSF(nm)
Base Number
RXR-DBD (5 ns)
RXR-DBD (10 ns)
D
RMSF (nm)
Base Number
VDR-DBD (5 ns)
VDR-DBD (10 ns)
E
RMSD (nm)
Time (ns)
WT1
WT1-DNA
F
Rg (nm)
Time (ps)
WT1
WT1-DNA
G
RMSF (nm)
Base Number
WT1 (5 ns)
WT1 (10 ns)
H
RMSF (nm)
Base Number
WT1-DNA (5 ns)
WT1-DNA (10 ns)

Supple. Fig. 2

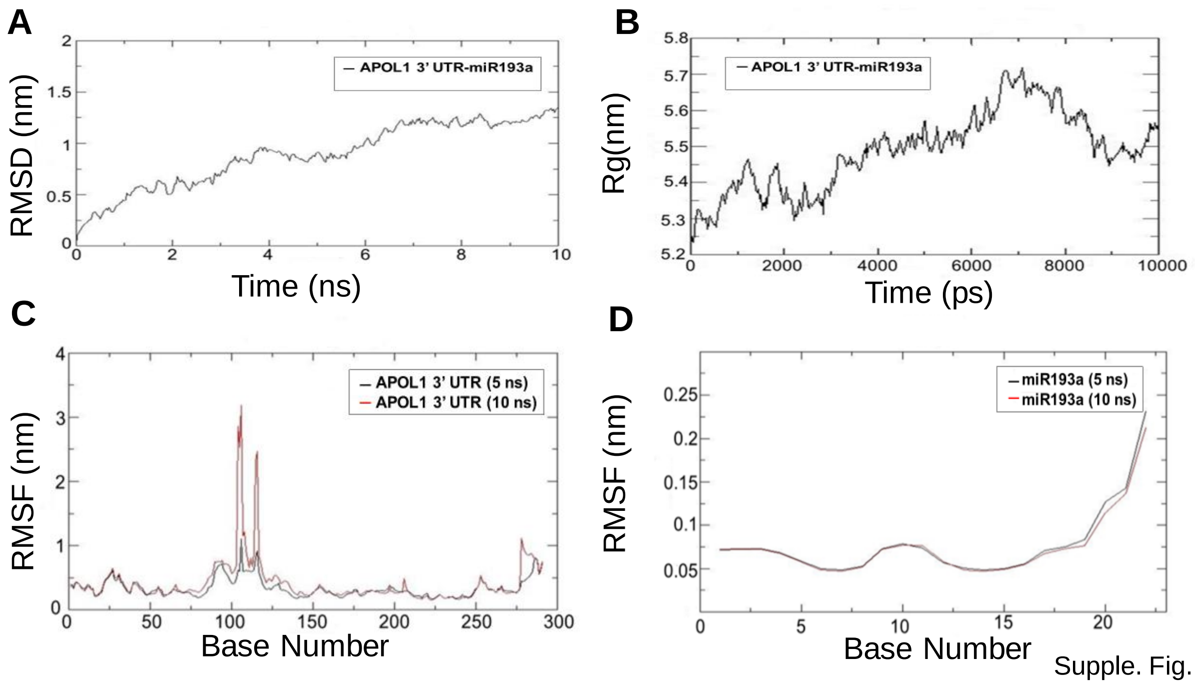

A
RMSD (nm)
Time (ns)
APOL1 3' UTR-miR193a
B
Rg(nm)
Time (ps)
APOL1 3' UTR-miR193a
C
RMSF (nm)
Base Number
APOL1 3' UTR (5 ns)
APOL1 3' UTR (10 ns)
D
RMSF (nm)
Base Number
miR193a (5 ns)
miR193a (10 ns)

Supple. Fig. 3

Supplementary Tables:

Table 1. Root mean square fluctuation (RMSF) values of interacting residues in DNA-Transcription complexes after 5 nano second (ns) and 10 nano second (ns) simulation

Complex	Residues	RMSF (5 ns)	RMSF (10 ns)
miR193a-YY1	Glu46	0.6022	0.6499
	Lys301	0.5794	0.5750
	Gly302	0.4441	0.4617
	Lys259	0.2831	0.2756
	Phe368	0.3686	0.4534
	Lys401	0.4175	0.5309
miR193a-Sox2	Tyr110	0.2107	0.3441
	Gln197	0.5970	0.6111
	Met236	0.4488	0.4431
	Ser251	0.4271	0.4911
	Arg264	0.4397	0.5312
miR193a-WT1	Arg321	0.3929	0.3645
	Lys332	0.3008	0.5032
	Lys386	0.4330	0.4619
	Thr443	0.6118	0.5796

Table 2. Root mean square fluctuation (RMSF) values of interacting residues in DNA-Transcription complexes after 5 nano second (ns) and 10 nano second (ns) simulation

Complex	Residues	RMSF (5 ns)	RMSF (10 ns)
miR193a-VDR-RXR			
RXR-DBD	Cys135	0.2732	0.4542
	Ala136	0.2759	0.3729
	Ile137	0.3969	0.4406
	Leu167	0.3514	0.3327
	Thr168	0.2537	0.2615
	Tyr169	0.2694	0.2962
	Thr170	0.2794	0.2907
	Lys175	0.3379	0.4122
	Asp176	0.3372	0.4685
	Leu178	0.4043	0.4460
	Ile179	0.4300	0.4573
	Tyr189	0.2304	0.2449
	Tyr192	0.2137	0.2365
	Gln193	0.2026	0.1943
	Lys194	0.2674	0.3073
	Leu196	0.2162	0.2185
	Ala197	0.2111	0.2251
	Met198	0.2318	0.2538
VDR-DBD	Arg18	0.2232	0.2352
	Asn19	0.2603	0.3080
	Val20	0.2029	0.2817
	Pro21	0.1817	0.2573
	Arg22	0.2077	0.2842
	Ile23	0.2055	0.2265
	Cys24	0.2600	0.2548
	Gly25	0.2542	0.2861
	Cys27	0.1237	0.1837
	Gly28	0.2124	0.2554
	Asp29	0.1394	0.1865
	Arg30	0.2051	0.2384
	Met39	0.3601	0.3926
	Arg67	0.1924	0.2322
	Ile68	0.2110	0.2774
	Thr69	0.2983	0.3006
	Lys70	0.3034	0.3092
	Asp71	0.2700	0.2813
	Asn72	0.3504	0.3440
	Arg83	0.2013	0.2089
